# Annual Meeting of the Alumni Association of the Baltimore College of Dental Surgery

**Published:** 1879-03

**Authors:** 


*Meeting of the Alumni Association of the Baltimore College of Dental Surgery.*—The annual meeting was held in the College Building on the morning of Commencement day, commencing at 11 o'clock.

Dr. S. J. Cockerille, of Washington, D. C., of the class of 53, presided, with Drs. H. B. Noble and W. A. Mills as Vice Presidents, and Dr. Thos. H. Davy as Recording Secretary. There was a very large number of Alumni present, and the meeting was a very interesting one.

The address to the Alumni was delivered by Professor F. J. S. Gorgas, and was paid marked attention. Dr. Gorgas impressed upon the minds of those who were about to enter upon the profession what is meant by self-knowledge; that duty consisted of honest, conscientious acts, and the reward, the good of mankind. He said that science was a jealous mistress, was progressive and researchive, that science and art were advancing with rapid strides, and that no profession had made more progress in so short a period as the profession of dentistry. We have a desire in this age to hasten things, and even colleges do not escape the mania. Yet he would warn the student not to attempt too much in too short a time; we must give proper time to all science. Dental science was a noble one, going hand in hand with others, and entitled to a position in the foremost rank, and though her progress had been rapid, it had been satisfactory, and was to-day a vast improvement on the dentistry of the past. In closing, Dr. Gorgas made mention of Dr. J. H. McQuillan, who died in Philadelphia on Wednesday last.

A committee of which Prof. J. B. Hodgkin was chairman, appointed to consider life insurance, reported it inexpedient to legislate at this time.

Resolutions offered by Dr. W. H. Hoopes on the merging of the Maryland Dental College with the Baltimore College of Dental Surgery were adopted, Dr. Henry Noble, of Washington, seconding the motion.

Resolutions of regret at the resignation of Dr. B. M. Wilkerson, Demonstrator of Operative Dentistry in the college were adopted.

The Committee appointed to award the prize for the best Thesis, on the conservative treatment of the dental pulp, consisted of Drs. W. H. Atkinson, C. S. Hurlbut, H. B. Noble and J. C. Green. The Committee to award the prizes for the best piece of plate-work, and the best gold filling in specimen tooth, were Drs. D. McFarland and W. W. Evans, of Washington, and Jas. F. Thompson, of Va.

Dr. C. S. Hurlburt, as chairman of the committee, then announced the prizes offered to the present graduates of the Baltimore College:

The first prize, set of Varney's pluggers, for the best thesis on Conservative Treatment of Dental Pulp, Andreas C. Ferrari, of Russia.

Second prize, "White" dental engine, for best plate work, Bolivar J. Quattlebaum, of South Carolina.

These prizes were offered by Dr. S. S. White, of Philadelphia.

Third prize, Harris' Medical and Dental Dictionary, for best filled specimen tooth, J. Armstead Colvin, of Virginia.

This prize was offered by Professor Gorgas.

Dr. Hurlburt then announced two prizes awarded by the Maryland College:

First prize, "White" dental engine, for best plate work, E. J. Parkison, of Maryland.

Second prize, set of Varney's pluggers, for best thesis on conservative treatment of dental pulp, Richard Grady, of Maryland.

These prizes were also offered by Dr. S. S. White, of Philadelphia.

The Alumni of the Maryland Dental College were then by vote made members of the Alumni of the Baltimore College of Dental Surgery.

An address by the President, S. J. Cockerille, on the aims and teachings of Dental Colleges followed, the speaker warning the graduates against the errors and charlatanry of the age.

A committee of five, consisting of Drs. Gorgas, Hodgkin, Cockerille, Hoopes and Davy, were appointed on the subject of an annual banquet.

A resolution was offered by Dr. James F. Thompson, and adopted, making provision for founding a library in connection with the College.

The annual election of officers was then held, resulting in the choice of Drs. C. S. Hurlburt, of Massachusetts, as President; J. F. Thompson, of Virginia, First Vice, and Henry Noble, of Washington, D. C., Second Vice President; W. H. Hoopes, of Maryland, Treasurer; W. A. Mills, of Maryland, Corresponding Secretary, and H. M. Schooley, of Washington, D. C., Recording Secretary.

Dr. Thomas S. Latimer was chosen as the orator of the dav for the next meeting of the alumni.

Dr. D. McFarland, of Washington, was elected an honorary member of the alumni, the college of which he was an alumnus having passed out of existence.

Dr. Cockerille on retiring from the chair, took occasion to thank the members for the aid, support, sympathy and courtesy which he had met with at their hands, which had made the position a pleasant one.

The newly elected officers were then conducted to the several chairs, the President, Dr. Hurlburt, returning thanks for the honor conferred, and in a short address alluded to the high standing of the College, the oldest in the world, and also of the high character of the men whom she had given to the profession.

Dr. Cockerille delivered a brief eulogy on the late Prof. Philip H. Austen, speaking of his life, character and superior attainments in the highest terms.

With the appointment of the Memorial Committee, consisting of Dr. S. J. Cockerille, Chairman; and Drs. T. H. Davy and R. B. Winder, the annual session of the alumni closed.

Banquet.—The Alumni banquet at the Howard House, which followed the Commencement exercises, was partaken of by about one hundred and fifty guests, and was a most elaborate affair, The "menu," which was gotten up in excellent style, consisted of twelve courses, served under the direct supervision of Mr. Ballard. On the cloth being removed the following toasts were given and responded to:

"The Coalition of the Colleges." Response by Hon. Judge G. W. Brown.

"Baltimore College of Dental Surgery." Response by Dr. S. J. Cockerille, of class of 1853.

"Dental Education." Response by Dr. J. F. Thompson, of Virginia.

"Alumni Association of the Baltimore College of Dental Surgery." Response by the President, Dr. C. S. Hurlburt, of Massachusetts.

"Our New Alumni, the graduates of '79." Response by Dr. John A. Millard, of France.

"The Medical Profession." Response by Prof. E. Cutter, of Boston.

"The Dental Profession." Response by Dr. R. B. Donaldson, of Washington, D. C.

"To the members of the Medical Profession who are teachers of Dental Students." Response by Prof. W. H. Atkinson, of New York.

"The Dental Profession of Europe." Response by Dr. A. C. Ferrari, of Russia.

"Dental Profession of New York City." Response by Dr. Parmley Brown.

"Dental Profession of Washington, D. C." Response by Dr. H. B. Noble, of Washington, D. C.

"Electricity in its Relations to Medicine." Response by Dr. J. J. Caldwell, of Maryland.

